# Association between continuous low-dose norepinephrine infusion and intraoperative hypotension burden in patients aged 80 years and older undergoing total hip arthroplasty: a retrospective cohort study

**DOI:** 10.3389/fmed.2026.1791081

**Published:** 2026-03-27

**Authors:** Xianya Zhao, Senlin Dong, Leilei Zhu, Weiwei Wu

**Affiliations:** 1 Department of Anesthesiology, The First Affiliated Hospital of Anhui Medical University (North District), Hefei, Anhui, China; 2 Anhui Public Health Clinical Center, Hefei, Anhui, China

**Keywords:** hypotension burden, intraoperative hypotension, norepinephrine, postoperative delirium, total hip arthroplasty

## Abstract

**Objective:**

To compare the associations of different intraoperative blood pressure management strategies with hypotension burden and postoperative recovery outcomes in patients aged 80 years or older undergoing total hip arthroplasty under general anesthesia.

**Methods:**

This single-center retrospective cohort study included patients aged ≥80 years undergoing total hip arthroplasty under general anesthesia. Patients were grouped according to intraoperative blood pressure management strategy: continuous low-dose norepinephrine (NE) infusion versus conventional reactive management. In the NE group, infusion was typically started immediately after induction and titrated within a usual range of 0.01–0.05 μg/kg/min according to continuous arterial pressure monitoring, overall hemodynamic trends, and clinical judgment, primarily for intraoperative blood pressure support. The primary outcome was intraoperative hypotension burden, quantified using the time-weighted average (TWA), area under the curve (AUC), and hypotension duration ratio (HDR) for MAP <65 mmHg. Secondary outcomes included postoperative delirium and postoperative length of hospital stay. Multivariable logistic regression was used to explore factors associated with postoperative delirium.

**Results:**

A total of 119 patients were analyzed (NE group, *n* = 56; control group, *n* = 63). Compared with conventional management, continuous low-dose NE infusion was associated with a lower intraoperative hypotension burden, including lower TWA of MAP <65 mmHg (0.24 [0.00–0.42] vs. 0.46 [0.25–0.83] mmHg; *p* < 0.001), as well as lower AUC and HDR (all *p* < 0.001). The NE group also had a lower incidence of POD (10.7% vs. 28.6%, *p* = 0.015) and a shorter postoperative length of hospital stay (7 [7–8] vs. 8 [7–9] days, *p* < 0.01). In multivariable analysis, the NE-based strategy remained independently associated with lower odds of POD after adjustment for age and history of stroke (adjusted OR 0.24; 95% CI 0.08–0.68; *p* = 0.007).

**Conclusion:**

In patients aged 80 years and older undergoing total hip arthroplasty, a continuous low-dose norepinephrine infusion strategy was associated with lower intraoperative hypotension burden and better postoperative recovery indicators. Given the retrospective observational design and potential residual confounding, these findings should be interpreted cautiously.

## Introduction

Patients aged 80 years and older undergoing major orthopedic surgery under general anesthesia are particularly vulnerable to intraoperative hemodynamic instability. Age-related physiological changes, diminished cardiovascular reserve, and a high burden of comorbidities markedly increase susceptibility to hypotension during anesthesia. In this population, cerebral autoregulation is frequently impaired, with a rightward shift and narrowing of the autoregulatory range, rendering cerebral perfusion more sensitive to even modest reductions in arterial pressure (1). Consequently, blood pressure levels traditionally considered acceptable may still be insufficient to maintain adequate organ perfusion in very elderly patients (2).

Intraoperative hypotension has been associated with adverse postoperative outcomes, including myocardial injury, acute kidney injury, and neurological complications (3). However, intraoperative blood pressure management strategies vary substantially in routine clinical practice. Approaches range from reactive vasopressor administration in response to hypotension to prophylactic continuous low-dose norepinephrine infusion initiated after anesthesia induction. Norepinephrine is increasingly favored because of its potent vasoconstrictive properties and minimal chronotropic effects at low doses, and it is thought to counteract anesthesia-induced vasodilation by restoring systemic vascular resistance (4). Despite its widespread use, comparative evidence regarding different blood pressure management strategies in very elderly patients undergoing orthopedic surgery remains limited.

Beyond immediate intraoperative effects, perioperative hemodynamic stability may also influence postoperative recovery. Postoperative recovery in elderly patients is a multidimensional process encompassing functional status, postoperative length of hospital stay, and neurocognitive outcomes. Among these, postoperative delirium (POD) is a clinically relevant and potentially modifiable complication that is associated with prolonged hospitalization and increased morbidity (5). Increasing evidence suggests that cumulative hypotension exposure, rather than isolated blood pressure thresholds, may play an important role in postoperative cognitive disturbances (6).

Therefore, this single-center retrospective cohort study aimed to compare different intraoperative blood pressure management strategies in patients aged 80 years or older undergoing total hip arthroplasty under general anesthesia. The primary objective was to evaluate their association with intraoperative hypotension burden, quantified using time-weighted average (TWA), area under the curve (AUC), and hypotension duration ratio (HDR). Secondary objectives included postoperative delirium and postoperative length of hospital stay. We hypothesized that, compared with conventional reactive management, a continuous low-dose norepinephrine infusion strategy would be associated with reduced intraoperative hypotension burden and more favorable postoperative recovery indicators.

## Methods

### Study design and ethical approval

This study was a single-center retrospective observational cohort study based on routinely collected clinical data. All anesthetic management strategies, including the use of norepinephrine infusion, were determined by the attending anesthesiologists as part of routine clinical practice, and no interventions were implemented beyond standard care for research purposes. The study protocol was approved by the Ethics Committee of the First Affiliated Hospital of Anhui Medical University (Approval No. PJ-YX2025-07). The requirement for written informed consent was waived due to the retrospective design and the use of anonymized medical records. Given the retrospective observational nature of the study, prospective trial registration was not required.

### Study population

**Figure 1:**
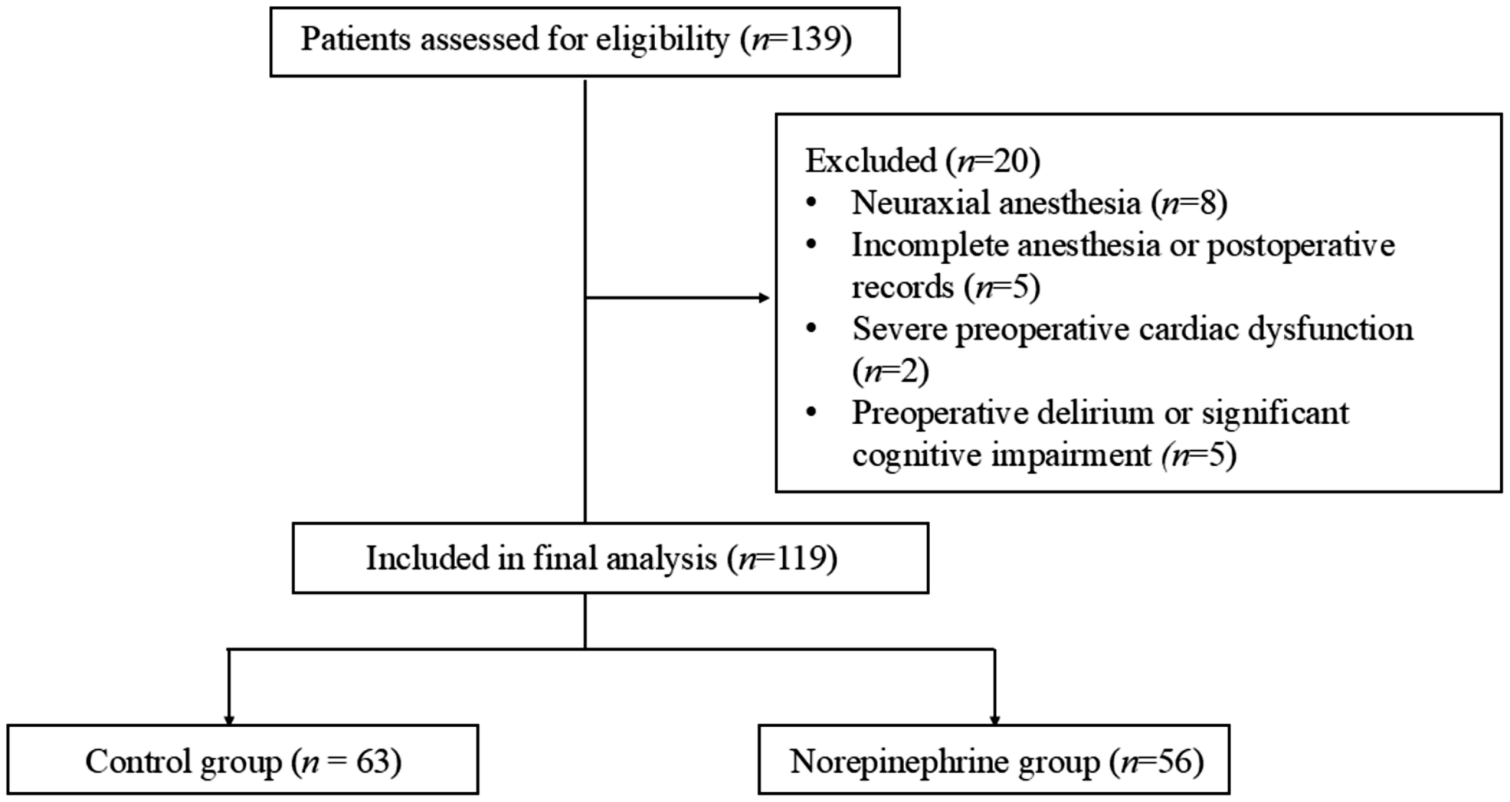
Flow diagram of patient selection.

This retrospective cohort study included elderly patients who underwent elective total hip arthroplasty under general anesthesia between January 2021 and October 2025 at the Department of Anesthesiology of the First Affiliated Hospital of Anhui Medical University (North District). A total of 139 consecutive patients aged 80 years or older were screened for eligibility. Patients were excluded if they received neuraxial anesthesia, had incomplete anesthesia or postoperative records, had severe preoperative cardiac dysfunction, or had documented preoperative delirium or significant cognitive impairment. After applying these exclusion criteria, 119 patients were included in the final analysis. No formal *a priori* sample size calculation was performed because of the retrospective cohort design; instead, the sample size was determined by the number of consecutive eligible patients available during the predefined study period. The patient selection process is shown in Figure 1.

### Intraoperative blood pressure management

Patients were categorized according to the intraoperative blood pressure management strategy used during routine clinical practice. The choice of strategy, including whether to use continuous low-dose norepinephrine infusion, was made at the discretion of the attending anesthesiologist as part of routine anesthetic management rather than according to a prespecified study protocol, standardized allocation rule, or predefined patient characteristics. Thus, treatment allocation in this retrospective cohort reflected variations in routine anesthetic practice rather than deliberate grouping based on patient risk profile. In patients managed with norepinephrine, infusion was typically started immediately after induction of general anesthesia and adjusted within a usual range of 0.01–0.05 μg/kg/min according to continuous invasive arterial pressure monitoring, overall hemodynamic trends, and clinical judgment. Although no rigid study-mandated titration algorithm was used, clinicians generally aimed to avoid sustained hypotension, particularly MAP <65 mmHg. Norepinephrine infusion was used primarily for intraoperative blood pressure support and was usually discontinued at the end of surgery. In a subset of patients, it could be continued during immediate transfer, in the post-anesthesia care unit (PACU), or into the early postoperative period at the discretion of the attending anesthesiologist according to hemodynamic status. In the control group, blood pressure was managed using conventional reactive strategies, including intermittent vasopressor administration and fluid boluses in response to hypotension.

### Intraoperative hemodynamic measurements

Mean arterial pressure (MAP) was continuously recorded via invasive arterial blood pressure monitoring. Intraoperative hypotension was defined as MAP < 65 mmHg, a commonly used absolute threshold in perioperative research, acknowledging the substantial heterogeneity in definitions reported in the literature (7, 8). Hypotension burden was quantified using three complementary metrics: time-weighted average (TWA), area under the curve (AUC) below 65 mmHg, and hypotension duration ratio (HDR) (9). In addition, the absolute duration of hypotension (minutes), defined as the cumulative time with a mean arterial pressure below 65 mmHg, was recorded as a descriptive intraoperative variable. Together, these indices capture both the depth and duration of hypotension exposure and provide a comprehensive assessment of cumulative hypotension.

### Postoperative outcomes

Postoperative delirium was identified based on routine clinical documentation in the medical records. A diagnosis of delirium was made by the attending physicians during postoperative ward rounds or nursing assessments within the first 7 postoperative days and was recorded in the electronic medical record. Psychiatric consultation was obtained when clinically indicated. Postoperative length of hospital stay was recorded. Other postoperative complications, including pulmonary infection, respiratory failure, acute kidney injury, atrial fibrillation, cerebral infarction, and stress ulcer, were collected. ICU admission was recorded separately.

### Statistical analysis

Continuous variables were assessed for normality and compared using the Student’s *t*-test or the Mann–Whitney *U* test, as appropriate. Categorical variables were compared using chi-square or Fisher’s exact tests. Multivariable logistic regression analysis was performed to explore factors associated with postoperative delirium. Variables were selected based on clinical relevance and prior literature. To reduce the risk of overfitting, only a limited number of covariates were entered into the multivariable model. Propensity score matching was not performed because of the limited sample size and the restricted availability of reliably recorded perioperative covariates in this retrospective dataset. Instead, baseline characteristics were compared directly between groups, and multivariable regression was used as an exploratory approach to adjust for potential confounding. Given the retrospective design and limited number of events, this multivariable analysis was considered exploratory and hypothesis-generating. A two-sided *p-*value < 0.05 was considered statistically significant.

## Results

Among the 139 consecutive patients screened, 119 met the inclusion criteria and were included in the final analysis (56 in the norepinephrine group and 63 in the control group). Baseline demographic characteristics and comorbidities were comparable between the two groups (Table 1). Regarding intraoperative management, phenylephrine was administered more frequently in the control group than in the NE group, whereas ephedrine use did not differ significantly between groups. In addition, the control group received a greater total intraoperative fluid volume than the NE group (950 [850–1,000] mL vs. 900 [800–1,000] mL, *p* = 0.038), while blood loss, crystalloid volume, red blood cell transfusion rate, and red blood cell transfusion units were similar between groups.

**Table 1:** Baseline and intraoperative characteristics of the study population.

Variable	Control group (*n* = 63)	NE group (*n* = 56)	*p*-value
Age (years)	85 (82–88)	86 (83–89)	0.136
Male sex, *n* (%)	16 (25.4)	18 (32.1)	0.416
BMI, kg/m^2^	20.5 (18.9–22.6)	21.0 (19.2–23.3)	0.230
ASA physical status, *n* (%)			0.204
II	8 (12.7)	12 (21.4)	
III	55 (87.3)	44 (78.6)	
Preexisting conditions, *n* (%)			
Hypertension	35 (55.6)	32 (57.1)	0.862
Coronary heart disease	10 (15.9)	8 (14.3)	0.809
Diabetes mellitus	13 (20.6)	11 (19.6)	0.893
History of stroke	30 (47.6)	30 (53.6)	0.717
Intermittent vasopressor use, *n* (%)			
Ephedrine	10 (15.8)	5 (8.9)	0.283
Phenylephrine	55 (87.3)	8 (14.3)	<0.001
Blood loss, mL	210 (200–250)	225 (200–260)	0.538
Total intraoperative fluid volume, mL	950 (850–1,000)	900 (800–1,000)	0.038
Crystalloid volume, mL	700 (650–750)	700 (638–750)	0.114
Red blood cell transfusion, *n* (%)	50 (79.4)	43 (76.8)	0.906
Red blood cell transfusion, units	1.0 (1.0–1.5)	1.0 (1.0–1.5)	0.315

Data are presented as median (interquartile range) or *n* (%). Continuous variables were compared using the Mann–Whitney *U* test, and categorical variables using the chi-square test or Fisher’s exact test, as appropriate. Percentages for individual intermittent vasopressors may not total 100% because some patients received more than one vasopressor.

NE, norepinephrine; ASA, American Society of Anesthesiologists; BMI, body mass index.

**Figure 2:**
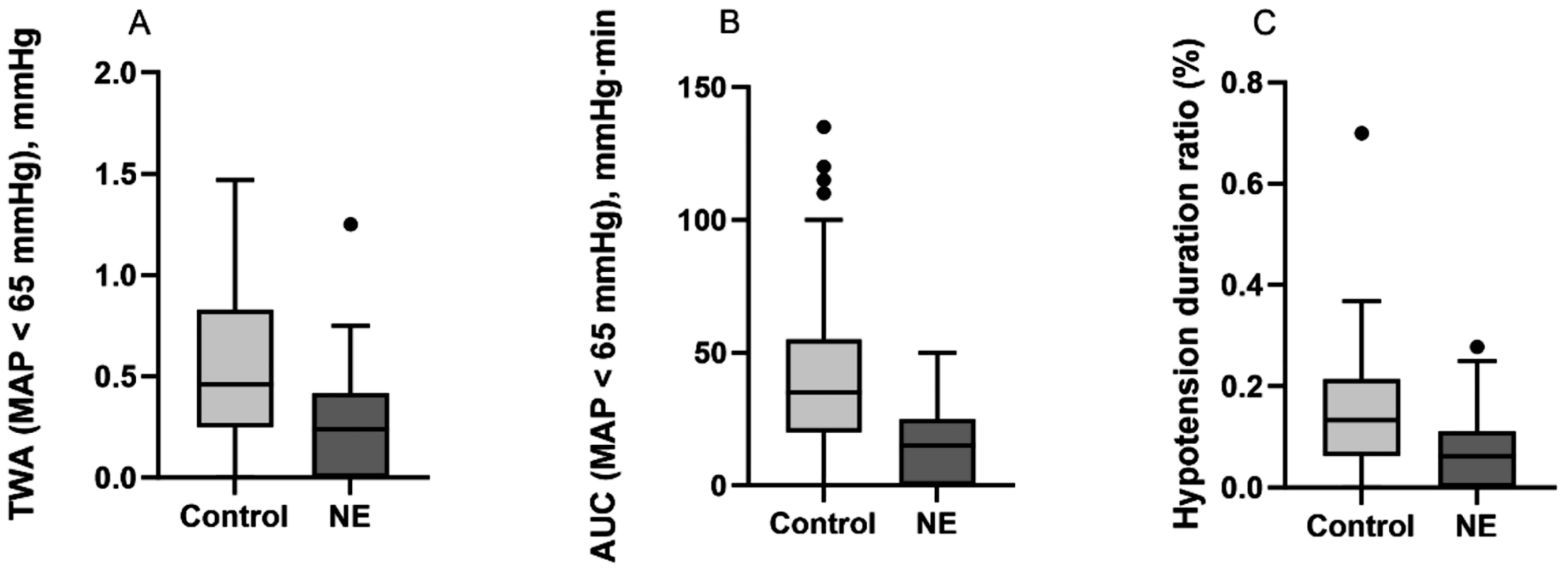
Comparison of intraoperative hypotension burden between groups. Data are presented as median (interquartile range). **(A)** Time-weighted average of mean arterial pressure (MAP) below 65 mmHg. **(B)** Area under the curve of MAP below 65 mmHg. **(C)** Hypotension duration ratio.

Patients in the NE group demonstrated significantly improved intraoperative hemodynamic stability compared with those in the control group. Specifically, the time-weighted average (TWA) of mean arterial pressure below 65 mmHg was significantly lower in the NE group than in the control group (0.24 [0.00–0.42] vs. 0.46 [0.25–0.83] mmHg; *p* < 0.001). The area under the curve (AUC) of MAP below 65 mmHg was also significantly reduced in the NE group (15 [0–25] vs. 35 [20–55] mmHg·min; *p* < 0.001), along with a lower hypotension duration ratio (HDR) (6.25% [0.00–11.11] vs. 13.33% [6.46–21.13]; *p* < 0.001). Intraoperative hemodynamic outcomes are summarized in Table 2 and Figure 2.

**Table 2:** Intraoperative hemodynamic outcomes.

Variable	Control group (*n* = 63)	NE group (*n* = 56)	*p-*value
Time-weighted average of MAP < 65 mmHg (TWA, mmHg)	0.46 (0.25–0.83)	0.24 (0.00–0.42)	<0.001
Area under the curve of MAP < 65 mmHg (AUC, mmHg·min)	35 (20–55)	15 (0–25)	<0.001
Duration of hypotension (min)	10 (5–15)	5 (0–6.25)	<0.001
Hypotension duration ratio (HDR, %)	13.33 (6.46–21.13)	6.25 (0.00–11.11)	<0.001

Data are presented as median (interquartile range).

MAP, mean arterial pressure; TWA, time-weighted average; AUC, area under the curve; HDR, hypotension duration ratio.

Postoperative outcomes are summarized in Table 3. The incidence of postoperative delirium was significantly lower in the NE group than in the control group (10.7% vs. 28.6%, *p* = 0.015). Patients in the NE group also had a significantly shorter postoperative length of hospital stay (7 [7–8] vs. 8 [7–9] days, *p* < 0.01). There was no significant difference between the two groups in the incidence of any postoperative complication (excluding POD) or ICU admission (all *p* > 0.05).

**Table 3:** Postoperative outcomes.

Variable	Control group (*n* = 63)	NE group (*n* = 56)	*p-*value
Postoperative delirium, *n* (%)	18 (28.6)	6 (10.7)	0.015
Postoperative length of hospital stay (days)	8 (7–9)	7 (7–8)	<0.01
Any postoperative complication (excluding POD), *n* (%)	16 (25.3)	10 (17.9)	0.337
Pulmonary infection	4 (6.3)	1 (1.8)	0.369
Respiratory failure	2 (3.2)	1 (1.8)	1.000
Acute kidney injury	5 (7.9)	4 (7)	1.000
Atrial fibrillation	1 (1.6)	1 (1.8)	1.000
Cerebral infarction	2 (3.2)	2 (3.6)	1.000
Stress ulcer	2 (3.2)	1 (1.8)	1.000
ICU admission	6 (9.5)	5 (8.9)	0.911

Data are presented as median (interquartile range) or number of patients (percentage). Patients may have experienced more than one postoperative complication; therefore, counts of individual complications do not sum to the total number of patients. Postoperative delirium (POD) and ICU admission were analyzed as separate outcomes and were not included in the composite complication outcome. POD, postoperative delirium.

In multivariable logistic regression analysis, the norepinephrine-based blood pressure management strategy remained independently associated with a lower risk of postoperative delirium after adjustment for age and history of stroke (adjusted OR 0.24; 95% CI 0.08–0.68; *p* = 0.007). A history of stroke was identified as an independent risk factor for postoperative delirium, whereas age was not significantly associated with POD in this cohort. These findings should be interpreted with caution given the limited number of events. Multivariable logistic regression results are presented in Table 4.

**Table 4:** Multivariable logistic regression analysis for postoperative delirium.

Variable	Adjusted OR	95% CI	*p-*value
Norepinephrine-based strategy (vs. control)	0.24	0.08–0.68	0.007
History of stroke	4.15	1.43–12.06	0.009
Age (per year)	1.02	0.96–1.09	0.529

Multivariable logistic regression analysis was performed to explore factors associated with postoperative delirium. Given the limited number of postoperative delirium events, this analysis should be interpreted as exploratory. OR, odds ratio; CI, confidence interval.

## Discussion

In this retrospective cohort study of patients aged 80 years and older undergoing total hip arthroplasty, a continuous low-dose norepinephrine infusion strategy was associated with a significantly lower intraoperative hypotension burden compared with conventional reactive blood pressure management. Importantly, the lower intraoperative hypotension burden observed in the norepinephrine group was accompanied by a lower incidence of postoperative delirium and a shorter postoperative length of hospital stay. Although causality cannot be inferred, these findings support an association between a continuous low-dose norepinephrine strategy and more stable perioperative hemodynamics in very elderly patients.

The clinical relevance of hypotension burden extends beyond traditional binary blood pressure thresholds. In very elderly patients, impaired cerebral autoregulation, characterized by a rightward shift and narrowing of the autoregulatory range, together with limited physiological reserve, may render cumulative hypotensive exposure particularly harmful (1). Metrics such as time-weighted average (TWA) and area under the curve (AUC) integrate both the depth and duration of hypotension and provide a more comprehensive characterization of intraoperative hemodynamic stress than isolated hypotensive episodes (2). In our cohort, the norepinephrine-based strategy achieved a markedly lower TWA compared with conventional management (0.24 [0.00–0.42] vs. 0.46 [0.25–0.83] mmHg; *p* < 0.001), along with significant reductions in AUC and hypotension duration ratio. This physiological context highlights the limitations of applying uniform blood pressure thresholds in very elderly patients and lends support to emerging concepts of more individualized blood pressure management aimed at preserving organ perfusion (10). The more frequent use of intermittent phenylephrine in the control group further supports the notion that patients managed without continuous norepinephrine infusion more often required reactive vasopressor support for hypotensive episodes. Interestingly, despite receiving a greater total intraoperative fluid volume, patients in the control group still experienced a higher hypotension burden. This finding is consistent with the possibility that, in patients aged 80 years and older, a reactive strategy based primarily on fluid administration and intermittent vasopressor boluses may provide less stable hemodynamic control than early continuous low-dose norepinephrine support. However, given the retrospective design of this study, this interpretation should be considered hypothesis-generating rather than definitive.

Our findings are consistent with a growing body of evidence linking intraoperative hypotension to adverse postoperative outcomes. Large observational studies have demonstrated associations between hypotension during noncardiac surgery and increased risks of myocardial injury, acute kidney injury, stroke, and mortality (3, 11, 12). More recent work has emphasized that the cumulative burden of hypotension, rather than isolated blood pressure thresholds, may better capture the clinical impact of intraoperative hemodynamic instability, particularly in older surgical populations (2). Proactive and individualized blood pressure management strategies, including early vasopressor use and anticipatory hemodynamic interventions, have been shown to reduce hypotension burden in selected cohorts (4, 13–17). In addition, real-world evidence from elderly surgical populations has shown that perioperative management strategies are closely associated with postoperative recovery profiles, including complication rates and length of hospital stay, underscoring the complexity of perioperative care in older patients (18). However, data focusing specifically on very elderly patients undergoing major orthopedic surgery remain limited. In this context, our study extends the existing literature by providing real-world evidence on the association between proactive hemodynamic stabilization and perioperative outcomes in patients aged 80 years and older undergoing total hip arthroplasty.

The rationale for using low-dose norepinephrine in this setting extends beyond simple blood pressure augmentation. Anesthesia-induced vasodilation frequently leads to relative hypovolemia and blood pressure variability, while excessive fluid administration in very elderly patients may increase the risk of cardiac decompensation. From a physiological perspective, norepinephrine counteracts anesthesia-induced vasodilation by restoring systemic vascular resistance, thereby contributing to more stable arterial pressure (4). Similar real-world observations have shown that baseline vascular regulation and perioperative vasoactive requirements substantially influence intraoperative hemodynamic stability in older patients (19). In addition, no significant differences were observed in the incidence of specific cardiac or renal events, such as atrial fibrillation or acute kidney injury, between the two groups (Table 3), indicating that no apparent increase in these postoperative complications was observed in the low-dose norepinephrine group in this cohort. These findings are consistent with prospective studies suggesting that low-dose norepinephrine infusion during anesthesia induction is feasible and well tolerated (20).

Although this study was not designed to elucidate mechanistic pathways, the parallel reduction in hypotension burden and postoperative delirium incidence is consistent with existing hypotheses linking cerebral hypoperfusion to neuroinflammatory processes and postoperative cognitive disturbances (21). In very elderly patients with impaired cerebral autoregulation, even modest or cumulative reductions in arterial pressure may compromise cerebral perfusion, potentially contributing to delirium development (22). Our findings should therefore be interpreted as hypothesis-generating rather than evidence of causality.

The multivariable logistic regression analysis further supports the plausibility of our observations. Prior stroke emerged as an independent risk factor for postoperative delirium (adjusted OR 4.15; 95% CI 1.43–12.06; *p* = 0.009), aligning with established clinical knowledge and supporting the face validity of the model (23). Large observational studies and systematic analyses have similarly identified pre-existing cerebrovascular disease as a major contributor to postoperative delirium risk, highlighting the multifactorial nature of this complication (5). This finding highlights the heightened neurovascular vulnerability of patients with pre-existing cerebrovascular disease and suggests that intraoperative blood pressure management may be particularly important in this high-risk subgroup, a possibility that merits further prospective investigation. Given the limited number of postoperative delirium events, these results should be interpreted cautiously.

Postoperative delirium is a multifactorial outcome, particularly in very elderly orthopedic patients with limited physiologic reserve. In addition to intraoperative hypotension burden and preexisting stroke history, other perioperative factors, such as perioperative hypothermia, anesthetic depth, timing of surgery, fluid management, opioid exposure, and preoperative cognitive status, may also influence the risk of POD. These considerations suggest that the association observed in the present study should be interpreted within a broader perioperative context rather than as evidence of a single isolated mechanism. Because several of these factors were not comprehensively available or could not be reliably quantified in this retrospective dataset, residual confounding cannot be excluded, and the adjusted odds ratios should be interpreted cautiously as measures of association rather than evidence of causality. This interpretation is also consistent with recent studies in high-risk older surgical populations, which support the multifactorial nature of postoperative neurocognitive outcomes and underscore the importance of comprehensive perioperative risk assessment in vulnerable older patients (24, 25).

Several limitations should be acknowledged. The retrospective single-center design limits generalizability and precludes causal inference. Some potentially relevant perioperative confounders, including anesthetic depth, perioperative temperature, detailed fluid management strategy, and preoperative cognitive status, were not comprehensively measured or adjusted for. Although several fluid-related variables were comparable between groups, total intraoperative fluid volume differed significantly, and its potential influence on postoperative outcomes cannot be excluded. Postoperative delirium was identified on the basis of routine clinical documentation rather than systematic screening with validated assessment tools, which may have led to underestimation of POD, particularly hypoactive delirium, as well as possible outcome misclassification. In addition, because propensity score matching was not feasible in this retrospective study, residual confounding and selection bias cannot be completely excluded. Despite these limitations, this study provides real-world evidence regarding perioperative blood pressure management in a highly vulnerable population that remains underrepresented in perioperative research.

## Conclusion

In very elderly patients undergoing total hip arthroplasty, a continuous low-dose norepinephrine infusion strategy was associated with lower intraoperative hypotension burden, lower postoperative delirium incidence, and shorter postoperative length of hospital stay. Given the retrospective observational design, these findings should be interpreted cautiously and confirmed in prospective multicenter studies.

## Data Availability

The raw data supporting the conclusions of this article will be made available by the authors, without undue reservation.
